# The Development of Heart Failure Electronic-Message Driven Tips to Support Self-Management: Co-Design Case Study

**DOI:** 10.2196/57328

**Published:** 2024-11-07

**Authors:** Caleb Ferguson, Scott William, Sabine M Allida, Jordan Fulcher, Alicia J Jenkins, Jo-Dee Lattimore, L-J Loch, Anthony Keech

**Affiliations:** 1 School of Nursing, Faculty of Science, Medicine, and Health, University of Wollongong, 33 Moore St, Liverpool, 2170, Australia, 61 (02) 8763 6000; 2 Centre for Chronic & Complex Care Research, Blacktown Hospital, Western Sydney Local Health District, Blacktown, Australia; 3 National Health and Medical Research Council Clinical Trials Centre, University of Sydney, Sydney, Australia; 4 Royal Prince Alfred Hospital, Sydney Local Health District, Sydney, Australia; 5 Baker Heart and Diabetes Institute, Melbourne, Australia; 6 Independent Consumer Co-Research Representative, Sydney, Australia

**Keywords:** heart failure, co-design, smartphone, app design, patient education, e-TIPS, electronic-message driven tips

## Abstract

**Background:**

Heart failure (HF) is a complex syndrome associated with high morbidity and mortality and increased health care use. Patient education is key to improving health outcomes, achieved by promoting self-management to optimize medical management. Newer digital tools like SMS text messaging and smartphone apps provide novel patient education approaches.

**Objective:**

This study aimed to partner with clinicians and people with lived experience of HF to identify the priority educational topic areas to inform the development and delivery of a bank of electronic-message driven tips (e-TIPS) to support HF self-management.

**Methods:**

We conducted 3 focus groups with cardiovascular clinicians, people with lived experience of HF, and their caregivers, which consisted of 2 stages: stage 1 (an exploratory qualitative study to identify the unmet educational needs of people living with HF; previously reported) and stage 2 (a co-design feedback session to identify educational topic areas and inform the delivery of e-TIPS). This paper reports the findings of the co-design feedback session.

**Results:**

We identified 5 key considerations in delivering e-TIPS and 5 relevant HF educational topics for their content. Key considerations in e-TIP delivery included (1) timing of the e-TIPS; (2) clear and concise e-TIPS; (3) embedding a feedback mechanism; (4) distinguishing actionable and nonactionable e-TIPS; and (5) frequency of e-TIP delivery. Relevant educational topic areas included the following: (1) cardiovascular risk reduction, (2) self-management, (3) food and nutrition, (4) sleep hygiene, and (5) mental health.

**Conclusions:**

The findings from this co-design case study have provided a foundation for developing a bank of e-TIPS. These will now be evaluated for usability in the BANDAIDS e-TIPS, a single-group, quasi-experimental study of a 24-week e-TIP program (personalized educational messages) delivered via SMS text messaging (ACTRN12623000644662).

## Introduction

Heart failure (HF) is a common, burdensome, and complex clinical syndrome that results in impairment of ventricular filling or ejection of blood to systemic circulation due to functional or structural heart abnormalities. The global prevalence of HF ranges between 0.4% and 6.8%, depending on the region [1]. In Australia, HF affects almost 1% of the population [2]. Morbidity and mortality rates are high, with approximately 50% to 65% of people with HF dying within 5 years of diagnosis [3]. Hospitalization and emergency department visits are common and costly, often attributed to poor self-care. Critical to addressing this is education, ensuring patients and their families or caregivers are educated, empowered, engaged, and equipped with the knowledge, skills, and capabilities to self-manage all facets of HF, including its risk factors and treatments.

The 2018 Australian Heart Foundation/Cardiac Society of Australia and New Zealand (CSANZ) Guidelines for the prevention, detection, and management of HF have recommended that targeted patient education is provided throughout the continuum of HF management [3]. Patients should be educated on their condition, symptoms (including exacerbation triggers, tracking, recognition, and management), therapies (including medications and nonpharmacological approaches), and management of possible complications, along with when to contact health care providers for assistance with symptoms, with clinical deterioration, or in case of an emergency [4]. An important challenge is low health literacy, which has been estimated to affect 59% of Australians [5]. The risk factors of low health literacy are older age, multimorbidity, immigrant status, low socioeconomic status, low education levels, and a person’s primary language being different from that of the available educational resources. People with low health literacy may lack access to or skills related to technology use [5].

Historically, patient education has been provided through a face-to-face approach, facilitated using printed or written materials and the use of video to support key messages [6]. COVID-19 has been a catalyst for the digital health revolution. In the last decade, we have seen a dramatically increased use of mobile health (mHealth), smartphone, tablet apps, and SMS text messaging to assess, educate, support, and interact with patients [7]. The engagement of end users (such as clinicians and people with lived experience) in the design of new mHealth HF solutions has been recommended to deliver a responsive evidence base that is relevant to those who will use such solutions and recommend their use [8]. Thus, the aim of this study was to partner with clinicians and people with lived experience of HF to identify the priority educational topic areas for the development of a bank of education electronic-message driven tips (e-TIPS) to support HF self-management and inform the delivery of these e-TIPS.

## Methods

### Study Design

**Figure 1.**
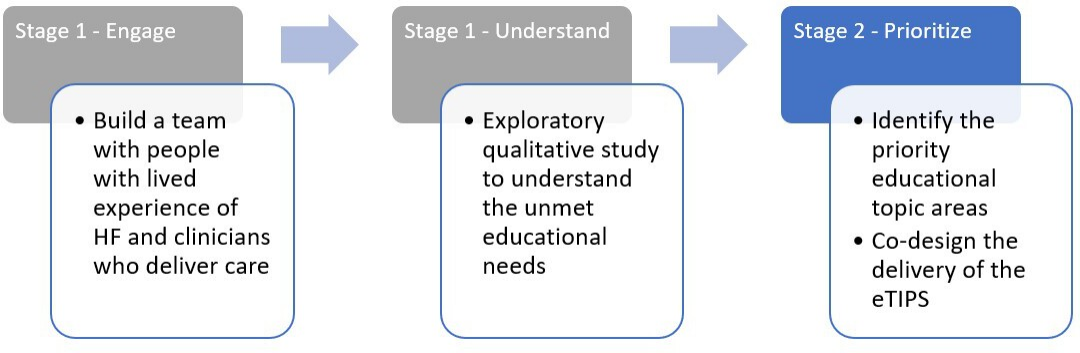
Co-design process. e-TIP: electronic-message driven tip; HF: heart failure.

We conducted 3 focus groups with cardiovascular clinicians and people living with HF and their caregivers, which consisted of 2 stages: stage 1 (an exploratory study to understand the unmet education needs of people living with HF) and stage 2 (a co-design feedback session to identify the priority educational topic areas and inform the delivery of the e-TIPS). This co-design case study was guided by the New South Wales (Australia) Agency for Clinical Innovation co-design principles and toolkit [9]. Co-design uses a staged process that adopts participatory and narrative methods to understand the experiences of receiving and delivering services, followed by people with lived experience and clinicians co-designing improvements or an intervention collaboratively. Refer to Figure 1 for the co-design process of this study. This paper reports the findings from stage 2 of the co-design process.

### Ethical Considerations

This study was designed and conducted as per the National Statement on Ethical Conduct in Human Research [10] and the Declaration of Helsinki 1975 as revised in 2000 [11]. The project was approved by the Sydney Local Health District (Royal Prince Alfred Hospital) Human Research Ethics Committee (approval number: X21-0484). Written and informed consent was obtained from all participants prior to participating in a focus group discussion. Audio data were transcribed by Pacific Transcription, an external, professional transcription service. Prior to transcription, participant identifiers were removed and replaced with a unique study participant ID. Data were stored on a secure password-protected network drive, only accessible to the project team.

### Sampling and Recruitment

We used a purposive sampling strategy to gain insights into the range of perspectives that need to be considered when developing e-TIPS for people with HF. In doing so, we sought out multidisciplinary clinicians working primarily in the cardiovascular specialty, people living with HF, and their caregivers. Participants were invited through list-service email distribution at participating hospitals (St. Vincent’s Hospital, Royal Prince Alfred Hospital, Concord Repatriation General Hospital, and Blacktown and Mount Druitt Hospital), which are primarily tertiary referral hospitals in Sydney, New South Wales, Australia. An invitation to participate was also emailed to professional societies including the Australian Cardiovascular Nursing College, CSANZ Cardiovascular Nursing Council, and the Allied Health Council of the CSANZ. Community-dwelling people with lived experience of HF and their caregivers were invited to participate through a study poster placed in the HF outpatient clinic of participating hospitals. Inclusion criteria were as follows: adults with a primary diagnosis of HF with reduced ejection fraction as per the National Heart Foundation of Australia and the European Society of Cardiology guidelines [12]; the ability to participate in face-to-face focus groups or via videoconference; and the ability to communicate and consent in English. The research team was diverse in gender, career stage, and disciplines including experts in HF, qualitative research methods, and a consumer co-researcher representative who was an adult living with HF.

### Data Collection

**Figure 2.**
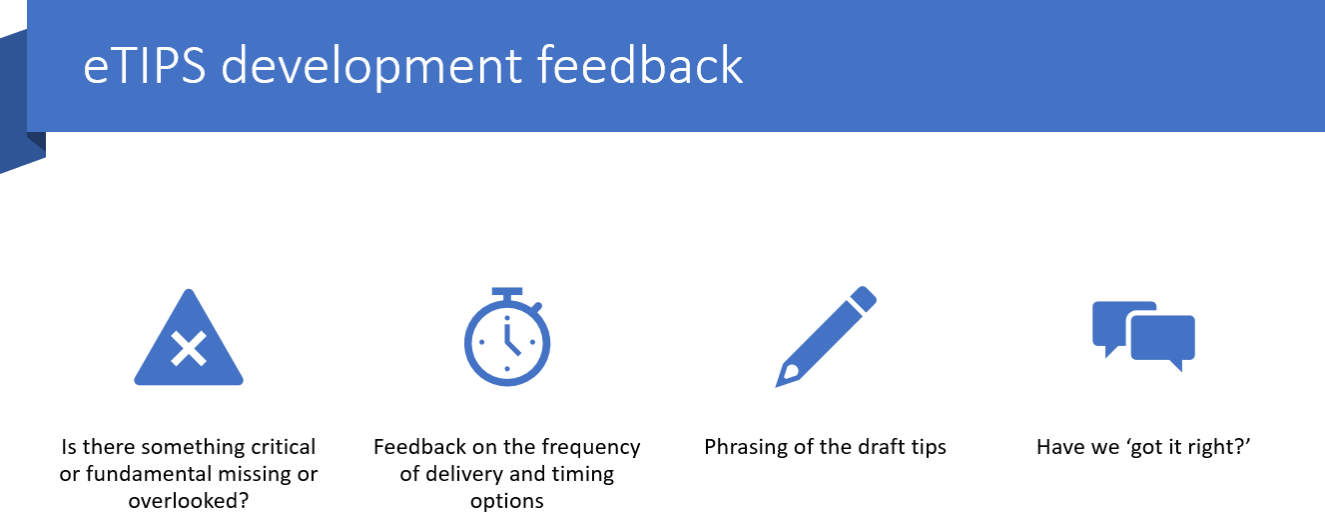
Stage 2 co-design feedback questions. e-TIP: electronic-message driven tip.

**Figure 3.**
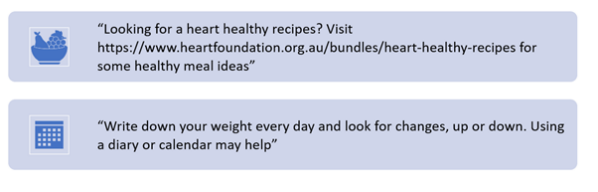
Draft electronic-message driven tips (e-TIPS).

We collected data through 3 focus groups conducted face-to-face or via videoconference between November and December 2022. Discussions were guided by a standardized interview guide, prepared by the study team (Multimedia Appendix 1). The questions in the interview guide were informed by the literature coupled with the research teams’ own experience of working with the patient group, and they were peer-reviewed by clinicians. Participants were provided with a short overview of key findings and topic areas from stage 1 and asked to provide feedback for alignment and improvement of eTIPS (refer to Figure 2 and Figure 3).

Expert facilitators (CF and AK) recorded the interviews using Zoom videoconferencing (Zoom Video Communications) or a digital audio recorder. The expert facilitators may have been known to some participants in the clinician groups; however, this facilitated rapport and communication. Following the interviews, the audio recordings were sent to a professional transcription service.

### Data Analysis

Two researchers independently reviewed and analyzed the transcripts to code for meaning units and generate categories, themes, and subthemes (SW and SA); disagreements were managed by a third reviewer (CF) to ensure the reliability and validity of the data. The researchers (SW and SA) were not known to the participants. Data analysis was finalized when no new codes or themes were discovered, and saturation was achieved. Inductive thematic analysis was undertaken between March and June 2023 with guidance by Braun and Clarke [13] using the computer software NVivo (version 12; QSR International; released in March 2020) to organize and code data [6]. A final report was written and used to provide findings for this paper. The Consolidated Criteria for Reporting Qualitative Research (COREQ) was used to guide the reporting of this study [14].

## Results

### Overview

We conducted 3 face-to-face or videoconferencing focus groups lasting between 73 to 91 minutes with 14 cardiovascular clinicians and 2 people with lived experience of HF. The clinician cohort comprised cardiologists and nurses with varying qualifications and expertise. The cardiologists were consultants engaged in providing either HF inpatient care or outpatient clinic-based care. The nurses were experienced clinical nurse specialists, clinical nurse consultants, or nurse practitioners. The patient cohort had a primary diagnosis of (HF with reduced ejection fraction) recruited from the outpatient HF clinic setting.

The first 2 focus groups (with 7 participants each) included 14 cardiovascular clinicians (11 nurses and 3 cardiologists). The subsequent focus group included 2 community-dwelling people with lived experience of HF (1 male, regional-based, and 1 female, metropolitan-based).

Two main themes were identified as follows: (1) key considerations for e-TIP delivery, and (2) relevant HF education modules are listed in Table 1.

**Table 1.** Themes and categories from thematic analysis.

Themes	Categories
Key considerations for e-TIP^a^ delivery	Timing of the e-TIPS Clear and concise e-TIPS Embedding a feedback mechanism Distinguishing actionable and nonactionable e-TIPS Frequency of the e-TIPS
Relevant HF^b^ education topic areas	Cardiovascular risk reduction Self-management Food and nutrition Sleep hygiene Mental health

a e-TIP: electronic-message driven tip.

b HF: heart failure.

### Key Considerations for e-Tip Delivery

#### Timing of the e-TIPS

Participants indicated that the e-TIP package can be adapted to time-based needs of treatment and management.

I think it’s also important to make the distinction because you take some tablets in the morning and not at night and vice versa.

[female lived experience expert, FG7]

#### Clear and Concise e-TIPS

Participants highlighted the importance of the wording and language used in the e-TIPS. This includes ensuring they are easy to understand with clear and concise wording.

This is just the copywriter coming out in me but, if you wanted to take your medication at the same time every day – and I just wonder whether it might make sense, rather than set a reminder to actually move to an active verb which is setting a reminder because it’s actually more embracing that proactive language can be more, one it’s more friendly but two it also shifts the responsibility if you like to the person reading the message rather than it being a bit hands off which is what set a reminder.

[female lived experience expert, FG7]

I think that you don’t want to make them too wordy either, do you. You don’t want them…being copious messages every day or every two days that takes up a whole screen and you need to scroll down to see the rest of the message. It’s hard to get it a bit succinct isn’t it.

[nurse, FG5]

#### Embedding a Feedback Mechanism

Clinicians and people with lived experience of HF reported the usefulness of providing question follow-ups and feedback loops to receive further information if desired.

I don’t know what how the tips are going to be and whether they come with a place to go for further information or whatever, it should be more frequently at the beginning. I don’t see two tips a week as being too onerous.

[nurse, FG6]

You also are going to have though a loop where people can say, hey, I’d like more information on A, B or C, so, that you can keep building out the tips.

[female lived experience expert, FG7]

You could just do it as an open-ended one with a, we’d welcome your input into other areas that we can cover with these tips and so you’ve just got that and so you don’t actually have the pressure of somebody responding and you could have an automated thank you for helping us build, continue to build…

[female lived experience expert, FG7]

#### Distinguishing Actionable and Nonactionable e-TIPS

It may be useful to provide context to patients if the e-TIPS are providing actionable information for self-management or solely providing information to increase their awareness of self-management concerns.

Well, are all your tips going to have a call of action in one way or another which is either increasing awareness or actually going out and doing something?

[female lived experience expert, FG7]

People with lived experience of HF should be involved in developing and revising the e-TIPS, as they have lived experience of HF.

e-TIPS could include information to assist patients to take action and escalate signs of deterioration.

Contact your doctor or nurse within 24 hours if these symptoms are being more noticeable, perhaps. That’s what’s in the [existing] heart failure action plan.

[cardiologist, FG5]

#### Frequency of the e-TIPS

The frequency of e-TIPS could be tailored and adjusted to the needs of the patients.

I would think frequently in the immediate post-discharge era and then diminishing with time post-discharge.

[cardiologist, FG6]

Weekly, maybe for the first four weeks and then less frequently after that.

[cardiologist, FG6]

### Relevant HF Education Topic Areas

#### Cardiovascular Risk Reduction

Links to existing resources can help provide patients with HF with useful self-management e-TIPS.

Yeah, and then the smoking cessation. Again, the same sort of thing, are links to things for smoking cessation?

[nurse, FG5]

People with lived experience of HF reported using their own personalized systems for monitoring and optimizing medication adherence, throughout the day, instead of using Webster packs. It could be a useful guide on what to do if they miss part of their daily routine.

Yeah, so I’ve actually got a process with the boxes where I take the boxes out of the cupboard and leave the lids open and then once I’ve taken it, I shut it and then I leave them out until night, and then I do the same thing and then they go back in the cupboard.

[female lived experience expert, FG7]

Useful e-TIPS could help patients understand what to do if they accidentally miss a dose or take too much medication. This could have serious consequences and may require immediate follow-up.

I think that’s actually one of the things I made a note of is, what happens in one of the self-management areas is what to do if you accidentally skip or miss?…Then the flip side of that is also what to do if you’ve accidentally taken or think you’ve taken more medication than you should have…do you find if something breaks that routine, that you have moments of confusion around whether or not you’ve taken your tablets?

[female lived experience expert, FG7]

Further to this, education on when to disclose the types of medications they take can be important.

There’s also another area around dental, like going – when you’re on blood thinners [anticoagulation] going to the dentist becomes a whole different experience and also going to the beautician.…Even just letting your dentist know if you’ve got a dental check coming up, letting your dentist know that you’re on blood thinners [anticoagulation].

[female lived experience expert, FG7]

#### Self-Management

Ensuring fluid and weight management advice is proportional to an individual’s needs, such as size and body mass.

Our threshold is plus two kilograms above what that would be considered their weight and similarly if they’re losing two kilograms. But that’s going to vary little bit according to how big or small the person is.

[cardiologist, FG6]

I try to focus a lot more on the symptoms and then recognising the symptoms so that then we can do something about it and maybe if it starts to become a problem and the weights going up and you are drinking two litres a day, then maybe we do need to pull back a bit. Because the alternative is you become breathless and that impacting on your quality of life. It can be quite a miserable life so trying to find some joy.

[nurse, FG6]

If you’ve got [caring for] a 42-kilo lady you can’t say, wait until your weight’s two kilos up and then call me, because that’s not going to cut it for her, because she’s little

[nurse, FG5]

Providing daily e-TIPS to help remind and reinforce the importance of their weight and fluid management was thought to be useful. This could include an e-TIP prompting the patient to record their weight daily and providing information to contact a clinician if changes are significant.

Is there any capacity in this service to do sort of scheduled messages where you could send that one first, write your weight down, and then later that day or the next day have a follow-up one, still in relation to weight saying, if your weight changes by x contact so and so.

[nurse, FG5]

I’ve got patients that record their weight very nicely and then come into hospital because it’s gone up 10 kilos, so recording the weight is important but they have to know what to do about it.

[cardiologist, FG5]

It is important to consider that not everyone has access to scales at home, and they might need advice on other signs and symptoms and additional advice on places where they can weigh themselves, such as their local pharmacy.

I believe, the question piecing together from people’s responses was training patients on fluid restriction and weights. One thing that we do find here is patients often can’t afford scales, they don’t have access to scales so giving them the typical metrics of two kilos in two days, it’s helpful for some but obviously it’s going to fall short for others. We have to have other strategies in place to help them manage that. Educating the patients on early triggers and linking that back to their [decompensation] events. Getting them to mentally revisit, what were the signs and symptoms that brought you into hospital? I’m sure others do this as well as part and parcel of the training, but particularly important for that patient group you don’t have access to scales or the bariatric patients that we see here that can’t afford the scales required to assess their weight. Then it’s about connecting them with places where they can go to check their weight and bearing in mind, that might only be weekly or fortnightly, sometimes it’s monthly.

[nurse, FG6]

An essential component of HF education should include guiding patients to recognize fatigue, exhaustion, and signs and symptoms of deterioration. It would also be necessary to provide tips for contacting a relevant clinician to escalate their concerns and get them to the appropriate care.

I think there’s also an element of trying to empower them [patients] to manage their own condition rather than do it completely passive.

[cardiologist, FG6]

They will often give us a call and we’ll either expedite into clinic or get them with their GP.

[nurse, FG6]

You can get advice from the physio[therapist] or the nurses that run the heart failure rehab in how far to push heart failure patients when they are walking or doing exercise. I always say to them, if you are walking but before you were sitting down for two minutes and then you can go back up and do a walk again. But this time, if you’re sitting down for five, 10 minutes, then that means that your exercise tolerance is a bit worse because of fluid overload. Yeah, I think it’s listening to their body. I think that it’s hard to but it’s knowing their signs and symptoms.

[nurse, FG6]

There are e-TIPS that could be developed to help manage thirst as part of their fluid management routine, with special considerations on seasonal factors that might result in different weather conditions, warranting adjustment in fluid restrictions.

I find I get up early in the morning when it gets a bit hot because I struggle with the water. I only have a bit of water, that’s the worst.…it’s only 1200mls. I have like 600 in the morning, 600 at night I find is the hardest trouble, when you’re out in the hot sun all day sweating and carrying on.…they just tell me I’ve got to have a certain amount and that’s it. So, on a hot day I’ll sneak a bit more in. It’s just – it’s terrible because you’re that dry around the mouth, stiff and sore.

[male lived experience expert, FG7]

I guess another point is that we haven’t really talked about seasonal variation, the advice you give in a hot summer might be quite different to a cold winter in terms of how much fluid because your [insensible] losses are generally going to be higher in the heat.

[cardiologist, FG6]

The e-TIPS could include strategies such as chewing gum, sucking on ice, and avoiding soft drinks to manage thirst.

I think that’s certainly one of the tips that we try to encourage is whether you guzzle on ice blocks, sucking on a little bit of an orange or lemon, a bit of citrus to make the mouth a little bit moist. Using a spray or the chewing gum that was mentioned, any of those tips. Obviously trying to give them an alternative.

[nurse, FG6]

We talked about sucking ice and chewing gum for people, so they don’t feel so dry as strategies if they’re on a very strict fluid restriction. Sometimes it’s possible to take more water as long as you take more Lasix [frusemide] as long as you can get it back out again then it’s not a problem to take more in especially in the hotter weather. Has anyone talked to you about that?

[female lived experience expert, FG7]

I keep asking the doctors for more water, but they won’t let me because I feel all stiff and sore now, I just get stiff and sore. If I need a bit more, I’ll just suck on a bit of ice at night-time.

[male lived experience expert, FG7]

If you can get the patient – if you could say, try and avoid soft drinks because they make you thirstier, because soft drink makes you thirsty and you get a lot of patients who will guzzle their soft drinks and you’re like, it’s not going to quench your thirst, ever.

[nurse, FG5]

Fatigue, overexertion, and exhaustion are significant issues for patients with HF to manage. e-TIPS could be developed that are focused on reducing exhaustion. This could include using a chair in the bathroom or kitchen to help manage fatigue and planning their day and week ahead of time.

People probably have different things on where’s best to go for this but if a patient’s asking you what they can and can’t do, because this came up quite a lot about what they’re allowed to do. What they can and can’t do and where to go for advice because we can give them the advice or make that suggestion of speak to your whatever. Do you have a place to go? What’s your go to for advice or do you say go and see an exercise physiologist or that the extreme? Or what’s your thoughts on that when we’re on the exercise topic?

[cardiologist, FG6]

I think it’s important to give them some tips for energy conservation as well…and particularly not just around their actual structured exercise for the day, but things like putting a chair in the bathroom or maybe if they’re in the kitchen chopping up vegetables, to use a chair so they’re not expending all their energy and they’re feeling better to do the more exciting things in their day or social activity.

[nurse, FG6]

It is common for patients with HF to express uncertainty about what they feel they can and cannot do physically; useful tips such as considering the factors involved with exercise locations could also be important.

I don’t know about you when you started to exercise but for me, I was concerned about pushing myself too hard and ending up back in a state versus wanting to go at it like a bull in a China shop, a bull at a gate and getting out there and getting healthy and working out what that right balance was took a little while.

[female lived experience expert, FG7]

One of the other things that I found out through trial and error was that walking even with a slight rise [walking on a slight elevation] was far more difficult than walking on flat ground. So, maybe somewhere in the exercise tips talking about starting with a walk on flat ground and building it up from there.

[female lived experience expert, FG7]

I think giving them the little tips about their day, maybe talk them through their routine and maybe if you can give them some specific advice as to what they can do. Whether it is the chair in the bathroom or planning their week ahead and appointments. It might be a good idea not to see your cardiologist and your respiratory physician in the one day because that might take too much out of you. Little things.

[nurse, FG6]

e-TIPS should also consider the climate and weather, in relation to the environment and time of day for exercise.

In summertime, I talk about what to do, about closing your house up, going to the local shopping centre where it’s got air conditioning, and strategies to keep cool.

[nurse, FG6]

Given that you’ve got something up about physical activity, you’d want to make sure that if they’re going to exercise, they exercise either early earlier in the day or later in the day when it’s hot.

[nurse, FG6]

#### Food and Dietary Choices

There is a priority in understanding food, vitamin supplements, and interactions with medications used for HF treatment and management.

When I was on warfarin and eating foods that I had no idea, because they didn’t show up in any of the charts were actually impacting on my ability to take the warfarin. So, even having that conversation around ginger.

[female lived experience expert, FG7]

About some medicines is it worth also including and vitamins don’t mix well together? Because a lot of people don’t realise the interaction of things, of vitamins and medication.

[female lived experience expert, FG7]

Education on how to identify “hidden” salts in food and drinks is essential. People with HF should also be mindful of micronutrients in processed foods, such as ready-made meals, condiments, or even juices. The e-TIPS should also consider different types of diet.

We generally tried to link salt to fluid restriction and that a lot of the products, a lot of the salt that people consume is hidden salt. I think trying to give a little bit of education about that because patients that largely follow a Mediterranean diet, just add a little sprinkle on their avocado or their tomato or a little bit in the pasta. They tend to generally be on a lower salt diet but it’s a lot of the processed food that people consume. Sauces, things that come out of a can or jar so we tend to not recommend alternatives. I prefer you add a little bit of salt but try to reduce the processed side of things. I try to have a fairly modest approach because for patients, it’s about quality of life and if they’re thirsty, you want them to be able to drink a little bit. But if they really go overboard because they’ve had a salty meal and they’re guzzling and then their feet are up the next day, try to pull back a little bit on the fluid intake the next day. Trying to be a bit moderate about your approach but linking the two that excessive salt can lead to a lot of thirst.

[nurse, FG6]

Same with, I think, tomato juice. I think that’s quite high in sodium.

[nurse, FG5]

So, the opportunities you take control through your diet, taking control through your nutrition…are all really helpful, positive ways of presenting the information too.

[female lived experience expert, FG7]

#### Sleep Hygiene

Some e-TIPS could be as simple as using a pillow to raise the head when sleeping or lying down to help with breathing difficulties, such as nocturnal dyspnea or orthopnea.

I think he’s pretty much covered all the themes. One more thing about lying down as well, I tend to be quite specific. If you’re lying flat, if you just use a pillow.

[nurse, FG6]

You might want to say an extra pillow under your head.…No, instead of put a pillow under your head going to sleep, saying putting a pillow under your head before sleeping can help.

[female lived experience expert, FG7]

#### Mental Health

Mental health changes can be a normal part of the HF illness trajectory. The difficulties around not being able to get out and about can contribute to mental health deterioration, including social isolation, loneliness, anxiety, and depression.

People can’t get out of the house sometimes to go and see them, so if we can go and see them that’s probably easier for the patient.

[nurse, FG5]

The following 2 quotes are part of a conversation between 2 people with lived experience of HF about their struggles with social isolation.

Yeah, well I’ve just got to keep moving mate otherwise it’s no good for me sitting around thinking.…Yeah, just done that. Like I said, sitting at home doing nothing is worse anyway.

[male lived experience expert, FG7]

I’m with you, does my head in if I can’t get out and do stuff.

[female lived experience expert, FG7]

Mental health manifestations can be associated with poor sleep hygiene, resulting in further problems. There are existing smartphone apps for mental health that are commonly used to manage emotional states. It was thought it could be helpful to reference these existing high-quality mental health resources, such as Beyond Blue [15] within the e-TIPS.

I know that we spoke about physical wellbeing but mental wellbeing, I think that’s one of the things that comes up for some of my patients about living with a chronic illness and how to find the positive side of things, even if it’s just one thing a day. So possibly calling a family member or a friend, or watching their favourite TV show.

[nurse, FG5]

Yeah, because there are a number of patients we’ve had over time where the heart failure started things and then the anxiety has really taken over as everything else.

[nurse, FG5]

For people who got the symptoms and anxiety and it’s all more part of the depression side of things, then they get the poor sleep because it becomes a bit of a…Vicious cycle. So ways to get out of that kind of cycle, and same with the on the anxiety thing. Will we have any links to any of the apps to help people with anxiety? The Beyond Blue ones and the various relaxation apps and things like that.

[nurse, FG5]

It could be a priority to provide resources to help differentiate between HF manifestations and mental health symptoms. It may also be important to highlight that providing mental health resources as part of an e-TIP package may not be a priority for e-TIP delivery.

You don’t want to have the patients thinking that everything that they’ve got is anxiety, they’ve got other symptoms happening as well. You need to have that level of education and of understanding what’s heart failure, what’s the anxiety on top of the heart failure…rather than being core to the actual tips you’re giving, it’s something they can hit off into if it’s something that’s useful for them, kind of a thing, rather than the main show.

[nurse, FG5]

## Discussion

### Principal Findings

This study provided a collaborative platform to facilitate the co-design of a bank of e-TIPS for HF self-management to trial in a future study (BANDAIDS e-TIPS) [16]. Clinicians and people with lived experience of HF were able to provide feedback through a series of facilitated virtual, iterative design focus groups. Feedback was sought around e-TIP messaging and content, phrasing and literacy, message frequency, delivery, and timing. Two main themes were identified as follows: (1) key considerations for e-TIP delivery and (2) relevant HF education modules. The recommendations for the educational content of e-TIPS focused on various aspects of self-management including weight management, medication adherence, dietary choices, and monitoring their health status and escalating to an appropriate service. Practical suggestions for self-improvement, such as tips to enhance mobility and exercise or plan appointments, were highlighted.

### Comparisons to Prior Work

Nurse-led education sessions have been shown to contribute to reduced rehospitalization and have demonstrated cost benefit [17]. However, these types of interventions can vary in quality, be resource-intensive to deliver, and demonstrate questionable sustainability [17]. Advances in new technologies call for new approaches to patient and caregiver education [18]. mHealth approaches appear to be acceptable and feasible [19]. However, a recent Cochrane review of mHealth-delivered education for people with HF found no evidence of mHealth interventions improving HF knowledge compared with usual care, and uncertainty around the evidence that mHealth improves self-efficacy, self-care, and health-related quality of life was reported. The review called for future research to robustly evaluate mHealth technologies for HF [20]. Recently, Chow et al [21], in the TEXT-ME randomized controlled trial among people with coronary heart disease, found that the use of lifestyle-focused SMS text messages improved low-density lipoprotein cholesterol, systolic blood pressure, and BMI; significantly increased physical activity; and significantly reduced smoking. mHealth digital apps have been reported to support self-management and medication adherence for blood pressure control, although the studies had a high risk of bias [22].

Patient digital literacy and health literacy are 2 separate and intertwined concerns with the follow-up of digital health interventions. A recent systematic review has revealed poor reporting of consumer involvement in co-design studies in chronic care [23]. Given that there is a greater prevalence of HF in people aged older than 65 years [24], there needs to be a large degree of consideration in the trial and implementation of digital health interventions for people living with HF. A recent systematic review of digital health intervention use in older patients with cancer has revealed simpler designs with a patient-perceived value from use can increase the likelihood of patient use and uptake in the long term [25]. Another review has indicated that readability and less complex descriptions are integral in producing digital health interventions for patients with either low digital or health literacy [26]. Other research has provided evidence that access to the internet and digital technologies is correlated with health literacy and outcomes [27,28], more notably in populations aged 65 years and older [29,30]. Recent research has suggested the divide in patient health outcomes for specific demographics could be a direct result of the digitalization of health services and health information provision [31,32].

### Future Directions

The development and delivery of e-TIPS aim to complement and augment existing HF management and care plans offered by health care providers in hospitals and community health services. This approach is recommended to be refined and enhanced by participants over time. Patients could provide their own tips through a coproduction system or citizen science approach to demonstrate valuable authentic partnerships with consumers and end users. This presents a workable model of digital education that may be replicable or tailored to the HF population. Results from this study have been used to inform the development of e-TIPS, which are being tested in the BANDAIDS e-TIPS, a single group, quasi-experimental trial of a 24-week e-TIPS program (personalized educational messages) delivered via SMS text messaging (ACTRN12623000644662) [16]. This will further determine the usability, impact, relevance, timing, and language of the messages for further customization and health-related quality of life at 6 months.

### Strengths and Limitations

This study has provided a strong foundation to inform the BANDAIDS e-TIPS, which will hopefully improve the feasibility, acceptability, and translation of the digital intervention.

This study has several limitations. First, the use of e-TIPS may not be feasible in patients with more advanced disease states, complex medical conditions, or low health literacy. Second, the low number of people with lived experience may relate to low health literacy and low digital literacy status of many of the potential participants. Third, the study sample was imbalanced and largely comprised of health care providers, compared with patients. This is not uncommon in co-design studies. Nonetheless, given that most health care providers interviewed specialized in HF, their combined experience treating patients with HF represents a substantive practical knowledge base to draw from. Last, the consumers engaged in this study may not be representative of the broader community and may limit the transferability of these findings. The need for future cultural adaptation of e-TIPS is acknowledged, including different language options.

### Conclusion

The educational content of e-TIPS focused on various aspects of self-management, including weight management; medication adherence; dietary choices; and monitoring signs and symptoms, including escalating symptoms of deterioration. Being clear, being concise, and tailoring the frequency of the e-TIPS to the patient’s needs were highlighted as important in their uptake. The findings of this co-design case study have laid the foundation for the development of a bank of e-TIPS currently being evaluated in the BANDAIDS e-TIPS study.

## Supplementary material

10.2196/57328 Multimedia Appendix 1 Interview guides.

10.2196/57328 Multimedia Appendix 2 BANDAID-EXPLORE investigators.

## Abbreviations

COREQ: Consolidated Criteria for Reporting Qualitative Research
CSANZ: Cardiac Society of Australia and New Zealand
e-TIP: electronic-message driven tip
HF: heart failure
mHealth: mobile health
