# Supplementary material for: The Development of Heart Failure Electronic-Message Driven Tips to Support Self-Management: Co-Design Case Study
Source: JMIR Cardio. 2024 Nov 7;8:e57328. doi: 10.2196/57328 (PMC11563649; doi:10.2196/57328)
Supplement: Multimedia Appendix 1 [file cardio-v8-e57328-s001.docx]

**Table S1. Patient focus group guide**

| INTERVIEW GUIDE |
| --- |
| 1. What is your experience of receiving patient education about heart failure?    1. How and when has this occurred? 2. What topics have been addressed in previous education about your heart failure? 3. What do you think is important information to know about your heart failure and its treatment and management? 4. What is important for you to know about your heart failure? 5. How and when would you like to receive patient education?    1. By whom and how often?    2. How would you like to be educated? 6. We are designing a new education program for heart failure, what do you think the priority topics for education should be? 7. Is there anything that is important to address about heart failure, that is currently not well addressed in patient education? |

**Table S2. Clinician focus group guide**

| INTERVIEW GUIDE |
| --- |
| 1. What is your experience of educating patients about their heart failure? 2. How are patients currently educated about their heart failure? 3. What do you feel are the core components of heart failure patient education? 4. What are the priority content areas that need to be addressed? 5. What are the most important topic areas to address? 6. How and when do you think patients like to receive education? 7. Do you feel there are any missing or unmet needs with current patient education? |
