# Supplementary material for: The Development of Heart Failure Electronic-Message Driven Tips to Support Self-Management: Co-Design Case Study
Source: JMIR Cardio. 2024 Nov 7;8:e57328. doi: 10.2196/57328 (PMC11563649; doi:10.2196/57328)
Supplement: Multimedia Appendix 2 [file cardio-v8-e57328-s002.docx]

**BANDAID-Explore Study Team Investigators**

Medical Research Future Fund – 2020 Cardiovascular Health Mission Grant (Ref: APP 2009251)

Digital solutions for heart failure best practice care

**Chief Investigators**

Professor Anthony Keech, University of Sydney & Royal Prince Alfred Hospital, Sydney

Dr Sean Lal, University of Sydney & Royal Prince Alfred Hospital, Sydney

Professor Peter Macdonald, University of New South Wales & St Vincent’s Hospital, Sydney

Professor Caleb Ferguson, University of Wollongong & Blacktown Hospital, Sydney

Mr Christopher Ryan, University of Melbourne

Professor Alicia Jenkins, University of Sydney

Dr Kathleen Dempsey, University of Sydney

Professor Clara Chow, University of Sydney & Westmead Allied Research Centre, Westmead Hospital, Sydney

Dr Rachel O’Connell, University of Sydney

Associate Professor Gary Kilov, University of Melbourne

**Associate Investigators**

Ms Rebecca Mister, NHMRC Clinical Trials Centre, University of Sydney

Professor Sandy Middleton, Australian Catholic University & St Vincent’s Hospital, Sydney

Dr Douglas Drak, University of Sydney

Professor Jo-Dee Lattimore, University of Sydney & Royal Prince Alfred Hospital, Sydney

Dr Andrzej Januszewski, University of Sydney

**Consumers**

Ms L-J L

Mr G B

**BANDAID-Explore Research Support**

Dr Sabine Allida, University of Wollongong & Blacktown Hospital

Mr Scott William, University of Wollongong & Blacktown Hospital

Ms Kaitlyn Griffin, Blacktown Hospital

Ms Portia Westall, University of Sydney

Ms Nicola Barrie, University of Sydney
